# Supplementary material for: Pre-meiotic 21-nucleotide reproductive phasiRNAs emerged in seed plants and diversified in flowering plants
Source: Nat Commun. 2021 Aug 16;12:4941. doi: 10.1038/s41467-021-25128-y (PMC8368212; doi:10.1038/s41467-021-25128-y)
Supplement: Supplementary file 3 — Description of Additional Supplementary Files [file 41467_2021_25128_MOESM3_ESM.pdf]

Description of additional supplementary information

Title: Supplementary Data 1

Description: miR11308 and miR2118/482 triggered 21-nt reproductive phasiRNAs in wild strawberry.

Title: Supplementary Data 2

Description: miRNAs identified in eudicots as triggers of reproductive 21-nt phasiRNAs.

Title: Supplementary Data 3

Description: PARE validation of miR11308 and miR2118/482 triggered 21-nt reproductive phasiRNAs in wild strawberry.

Title: Supplementary Data 4

Description: miR2118/482 and miR11308 triggered 21-nt phasiRNAs in rose.

Title: Supplementary Data 5

Description: miR2118/482- and miR14051-triggered 21-nt phasiRNAs in columbine.

Title: Supplementary Data 6

Description: PARE validation of miR2118/482- and miR14051-triggered 21-nt phasiRNAs in columbine.

Title: Supplementary Data 7

Description: miR2118/482-triggered 21-nt phasiRNAs in flax.

Title: Supplementary Data 8

Description: Enrichment analysis of 21-nt reproductive pathway genes in four eudicots.

Title: Supplementary Data 9

Description: Nomenclature of Argonaute-encoding genes.

Title: Supplementary Data 10

Description: Nomenclature of Dicer-like (DCL) genes.

Title: Supplementary Data 11

Description: Nomenclature of RDR-encoding genes.

Title: Supplementary Data 12

Description: Probes used for in situ hybridizations.

Title: Supplementary Data 13

Description: Public sRNA sequencing data used in this study.
